# Transfusion Practice in Elderly Surgical Patients in Germany—A Secondary Data Analysis of over 21 Million Patients over a Decade

**DOI:** 10.3390/medicina62071332

**Published:** 2026-07-10

**Authors:** Lea Valeska Blum, Thomas Jasny, Benjamin Friedrichson, Armin Niklas Flinspach, Vanessa Neef, Kai Zacharowski, Jan Andreas Kloka

**Affiliations:** Intensive Care Medicine and Pain Therapy, Department of Anesthesiology, University Hospital, Goethe University Frankfurt, 60590 Frankfurt, Germany

**Keywords:** blood transfusion, anemia, elderly, secondary data analyses, perioperative care

## Abstract

*Background and Objectives*: The aging population poses increasing challenges for perioperative care, particularly with respect to blood transfusion practices. This study evaluated transfusion patterns, associated risk factors, and outcomes among surgical patients aged 65 years and older. *Materials and Methods*: All hospitalized surgical patients in Germany between 1 January 2013, and 31 December 2022, were included. Patients aged ≥ 65 years were categorized as transfused (having received red blood cells (RBCs)) or non-transfused. Patients with overlapping procedures or unknown surgical disciplines were excluded. Comorbidities, anemia, bleeding, complications, and blood product use were analyzed using ICD and OPS codes. *Results*: A total of 21,143,317 surgical patients aged ≥ 65 years met the inclusion criteria; 2,640,608 (12%) received at least one RBC transfusion. The median age was 79 (73–85) years overall, 79 (73–85) years among transfused patients, and 76 (70–81) years among non-transfused patients. Women accounted for 55.0% of cases. The highest median Elixhauser comorbidity score was observed in patients aged 80–84 years and was significantly higher in transfused patients. Essential hypertension was the most common risk factor, while acute kidney injury was the most frequent complication. Length of stay, ventilation hours, and ICU admission rates decreased with increasing age. Patients aged 65 years and older had an odds ratio of 3.99 (3.98–4.00) for receiving a transfusion. *Conclusions*: In this observational study of surgical patients aged ≥ 65 years, the risk of receiving an RBC transfusion increased with age, whereas length of stay, ventilation time, and ICU admission rates decreased. These findings support individualized perioperative management strategies, emphasizing the appropriate evaluation and treatment of anemia and the rational use of blood products in older adults.

## 1. Introduction

With life expectancy rising worldwide, clinicians are encountering a growing population of elderly surgical patients. This demographic presents specific challenges due to a higher prevalence of comorbidities, reduced physiological reserves, and increased vulnerability to complications, all of which require tailored perioperative management and careful risk assessment. Additionally, significant differences in physical and mental fitness among patients of the same age highlight the need for a deeper understanding of elderly patients and the identification of specific characteristics and risk factors to enable personalized therapy. There is no clear definition of an “elderly” patient, as there is a wide range from fit to multimorbid patients of the same age. To reflect this multidimensionality of chronological age, frailty is a common surrogate parameter. Frailty captures functional and physiological deficits across multiple systems, such as muscle strength and cognitive function. In a meta-analysis, Vermeiren et al. described frailty as a predictor of negative health outcomes, with a 1.8- to 2.3-fold increase in the risk of mortality [1]. The German Society of Geriatrics likewise does not define a clear age threshold. In their guideline “Comprehensive Geriatric Assessment (CGA) for hospitalized patients,” they define a target population of patients aged 65 years and older [2]. Preoperative anemia is a strong predictor for perioperative red blood cell (RBC) transfusion, higher complication rates, and increased mortality [3]. In elderly patients, RBC-transfusions are particularly associated with higher complication and mortality rates [4,5,6]. A study by Blum et al. demonstrated that increasing age is an independent risk factor for complications, longer hospital stays, in-hospital mortality, and, in particular, the need for RBC transfusion [7]. In recent years, there has been a major focus on the risks and treatment of preoperative anemia and the avoidance of unnecessary blood transfusion. Research has shown that older patients suffer more frequently from anemia [8,9]. Deviating transfusion thresholds are being investigated [10], and transfusion thresholds for orthopedic patients over 65 years have been modified in the German guidelines [11]. Transfusion futility thresholds that take frailty into account are also discussed in the literature [12,13]. This study provides an overview of blood transfusions in elderly surgical patients, examining their demographics and associated complications over 10 years in Germany. The study aims to describe blood transfusions in elderly patients over time and to determine whether changes in transfusion practice can be observed over time, particularly considering the revisions to German transfusion guidelines and increasing awareness of the heterogeneity of aging as the population of older patients continues to grow.

## 2. Materials and Methods

### 2.1. Availability of Data

In Germany, hospitals are legally required to report relevant diagnoses using the International Statistical Classification of Diseases and Related Health Problems (ICD) codes and the International Statistical Classification of Procedures (OPS) codes [14] (Table A1). According to §21 of the German Hospital Finance Law (KHG), all hospitals must submit anonymized data to the Institute for Hospital Remuneration (InEK) for the ongoing development of the Diagnosis-Related Group (DRG) system. These data are subsequently transferred by InEK to the Federal Statistical Office (DESTATIS), where they are made accessible to researchers under a signed data use agreement. The reporting of ICD-10 and OPS codes, along with the dependency of reimbursement on these data, ensures comprehensive recording of all treatments in German hospitals. The data are stored by the German Federal Statistical Office. Individual patient and hospital identifiers were not accessible to the authors. As the data are anonymized by the German Federal Statistical Office, the General Data Protection Regulation (GDPR) does not apply. Consequently, the Ethics Committee of the University Hospital Frankfurt waived the need for approval and informed consent (Chair: Prof. Dr. Harder, Ref: 2022-766). Data were collected in a structured and representative manner according to the Declaration of Helsinki and the STROBE guidelines.

### 2.2. Inclusion Criteria

**Figure 1:**
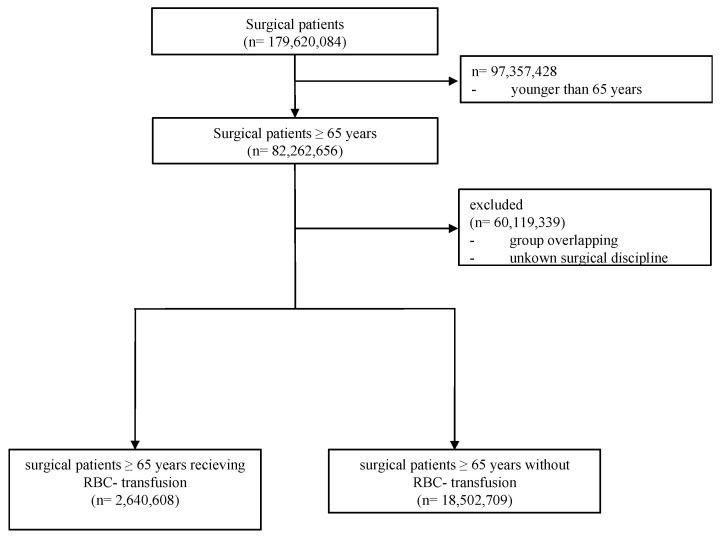
Flowchart.

Although there is no clear definition of “elderly,” the most common age cutoff reported in the literature is 65 years. We used the Youden index to determine the age threshold for transfusion using data from patients undergoing surgery between 1 January 2013, and 31 December 2022. The analysis suggested a cut-off value of 68 years (Figure 1). Therefore, 65 years seemed to be a reasonable cutoff value for the purpose of our analysis. All hospitalized surgical patients aged ≥65 years in Germany between 1 January 2013, and 31 December 2022, who underwent general surgery, gynecologic surgery, cardiac surgery, thoracic surgery, oral and maxillofacial surgery, neurosurgery, trauma and orthopedic surgery, urologic surgery, or vascular surgery, were included in the analysis.

Patients were categorized into two groups: those who received RBC transfusions and those who did not (transfused and non-transfused, respectively). Patients who underwent surgeries in multiple disciplines were excluded. For the estimation of the odds ratio for transfusion in patients aged ≥ 65 years, patients aged < 65 years were used as the reference group for this specific analysis, including those otherwise excluded from the primary cohort.

### 2.3. Definitions and Data Acquisition

All listed items were grouped and analyzed according to whether patients received RBC transfusions (transfused) or did not receive RBC transfusions (non-transfused). Diagnoses were coded according to the 10th revision of the International Classification of Diseases (ICD-10), and procedures were coded according to the International Classification of Procedures in Medicine (version 2020). The assignment of collected data, as well as the corresponding OPS and ICD-10 codes for each procedure and disease, can be found in Table A1. The Elixhauser Comorbidity Index is a scoring system that quantifies a patient’s comorbidity burden based on 31 conditions and is used to predict hospital outcomes such as mortality and length of stay.

### 2.4. Statistical Analysis

Categorical variables are expressed as absolute numbers and percentages. Continuous variables were tested for normality. All continuous variables considered (age, length of stay (LOS), and mechanical ventilation) were non-normally distributed. Hence, continuous variables were presented as medians with the 25th and 75th percentiles. Given the large sample size, even small between-group differences may reach statistical significance and should be interpreted in the context of their clinical relevance. Predefined comorbidities, RBC transfusion, anemia, bleeding, complications, and blood product usage were analyzed using their respective ICD and OPS codes, as defined in Table A1. Multiple logistic regression models were conducted. In the first model, the probability of RBC transfusion in patients older than 65 years was estimated. In the second model, the probability of RBC transfusion among patients over 65 years was estimated, adjusting for age groups and the effect of sex, Elixhauser score, ICU-admission and anemia. Data handling was performed using Excel 2019 (Microsoft Corp., Redmond, WA, USA), and statistical analyses were conducted using SAS (Version 9.4M6, SAS Institute Inc., Cary, NC, USA).

## 3. Results

Between 1 January 2013, and 31 December 2022, 82,262,656 patients aged ≥ 65 years underwent surgery, of whom 60,119,339 were excluded because of overlapping surgical disciplines or unknown surgical disciplines. In total, 21,143,317 patients were analyzed, of whom 2,640,608 (12%) received an RBC transfusion (Figure 1).

The proportion of female patients increased continuously with age (Table 1) and was higher in the transfused group than in the non-transfused group (55%, n = 1,453,376 vs. 50%, n = 9,310,912) (Table 2). In the transfused group, the median age was 79 (73, 85) years, while in the non-transfused group, it was 76 (70, 81) years. In both groups, the largest age demographic was 75–79 years, comprising 23% (n = 607,324) of transfused patients and 24.7% (n = 4,564,518) of non-transfused patients (Table 2). Because a single patient may be diagnosed with multiple types of anemia, it was not possible to determine an overall anemia rate. The most common form of anemia, classified as “any other form of anemia,” was observed in 16% (n = 3,298,924) of all patients. It was present in 90.5% (n = 2,389,159) of transfused patients and 4.9% (n = 909,765) of non-transfused patients (Table 2). The overall rate of iron deficiency anemia was 2.0% (n = 484,596). It was present in 7.5% (n = 199,161) of transfused patients, with the highest percentage observed among patients aged 80–84 years (8.2%, n = 49,907), and in 1.5% (n = 285,435) of non-transfused patients (Table 2).

Comorbidity burden, as reflected by the Elixhauser score, was broadly comparable across age groups, although small distributional differences reached statistical significance due to the large sample size. Among age groups, the highest median Elixhauser score was observed in patients aged 80–84 years (11 (5, 17)). Transfused patients aged 65–69 years had the highest intensive care unit (ICU) admission rate (22.8% (n = 74,673)). This rate decreased progressively with age, reaching 17% (n = 7840) in patients aged 95–110 years. Ventilation time followed a similar trend, declining from 95 (25, 338) hours in patients aged 65–69 years to 24 (7, 61) hours in those aged 95–110 years. LOS varied by approximately 2 h among patients aged 65–84 years and decreased in older patients (Table 1).

Additionally, the percentage of patients admitted to the ICU was nearly four times higher in the transfused group than in the non-transfused group (19.7% vs. 5%). Both LOS and duration of mechanical ventilation were approximately twice as long in transfused patients, with a median LOS of 363 (238, 600) hours in transfused patients compared with 150 (74, 263) hours in non-transfused patients, and a median ventilation time of 70 (20, 260) hours versus 30 (8, 112) hours (Table 1 and Table 2).

### 3.1. Risk Factors

**Figure 2:**
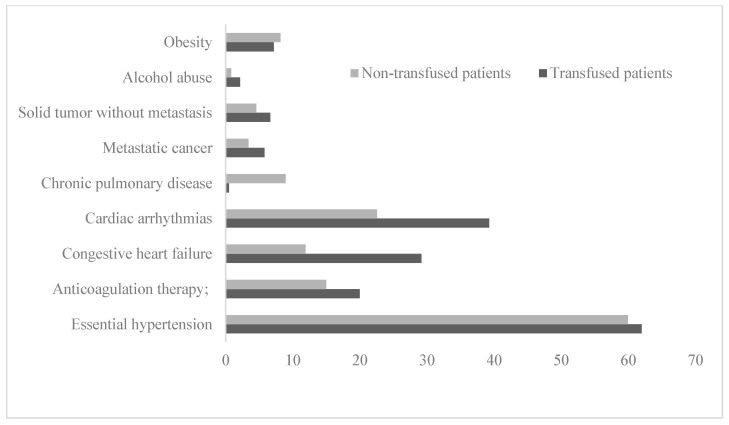
Proportions of risk factors in transfused and non-transfused elderly hospitalized surgical patients in Germany in percentages (2013–2022).

Among both transfused and non-transfused patients, the most common perioperative risk factor was essential hypertension (62%, n = 1,636,533 in transfused patients; 59.9%, n = 11,088,807 in non-transfused patients), followed by cardiac arrhythmias (39.2%, n = 1,035,781 in transfused patients; 22.6%, n = 4,173,921 in non-transfused patients) (Figure 2). Additionally, rates of anticoagulation therapy, congestive heart failure, cardiac arrhythmias, solid tumors without metastasis, metastatic cancer, and alcohol abuse were higher among transfused patients than among non-transfused patients, with the largest difference observed in congestive heart failure (29.2%, n = 769,881 in transfused patients vs. 11.9%, n = 2,201,409 in non-transfused patients).

In contrast, non-transfused patients had higher rates of chronic pulmonary disease and obesity, with the greatest difference observed in chronic pulmonary disease (0.5%, n = 327,662 in transfused patients vs. 8.9%, n = 1,646,280 in non-transfused patients) (Figure 2).

Across age groups, the incidence distribution remained consistent. Essential hypertension was the most common risk factor in all age groups, with the highest rate observed among patients aged 90–94 years (64.7%, n = 126,802). The rate of cardiac arrhythmias peaked in patients aged 85–89 years, while congestive heart failure and anticoagulation therapy rates were highest in those aged 90–94 years. By contrast, the incidence of chronic pulmonary disease, obesity, metastatic cancer, solid tumors, and alcohol abuse decreased with increasing age (Table 1 and Table A2).

### 3.2. Complications

**Figure 3:**
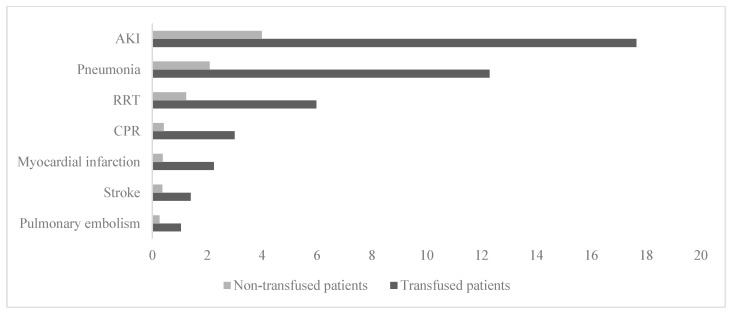
Proportions of complications in transfused and non-transfused elderly hospitalized surgical patients in Germany (2013–2022) in percentages. CPR: Cardiopulmonary Resuscitation. RRT: Renal Replacement Therapy. AKI: Acute Kidney Injury.

The rate of observed complications was higher in transfused patients: pulmonary embolism (PE) (1.0% vs. 0.3%), stroke (1.4% vs. 0.4%), myocardial infarction (2.2% vs. 0.4%), cardiopulmonary resuscitation (CPR) (3% vs. 0.4%), renal replacement therapy (RRT) (7% vs. 1.2%), pneumonia (12.3% vs. 2.1%), and acute kidney injury (AKI) (17.7% vs. 4%). The most common observed complication was AKI, which also showed the largest difference in complication rates between the groups: 17.7% (n = 466,023) in transfused patients and 4% (n = 738,740) in non-transfused patients. This was followed by pneumonia, with rates of 12.3% (n = 324,446) in transfused patients compared with 2.1% (n = 386,876) in non-transfused patients (Figure 3). For detailed rates of additional complications, see Table 2. Across age groups, the incidence distribution remained consistent. AKI was the most common complication in all age groups, with the highest rate observed in patients aged 80–84 years (18.4%, n = 111,348), followed by pneumonia, with the highest rate observed in patients aged 65–69 years (13.6%, n = 44,402) (Figure 3, Table A3).

### 3.3. Detailed Transfusion Practice in Elderly Patients

Among the 2,640,608 transfused patients, 88% (n = 2,323,148) received between 1 and 5 RBC units; 9.3% (n = 244,282) received 6 to 10 RBC units, and 1.8% (n = 46,684) received 11 to 15 RBC units. For higher numbers of RBC units, see Table A4. Additionally, 31.8% (n = 840,140) received RBCs prior to surgery. Among the transfused patients, 4.0% (n = 106,564) also received platelets, 4.0% (n = 233,675) received fresh frozen plasma (FFP), 5.8% (n = 152,391) received prothrombin complex concentrate (PCC), and 3.6% (n = 96,010) received fibrinogen. Of all transfused patients, 2.9% (n = 76,005) underwent massive transfusion (defined as more than 5 RBCs) (Table 3). There is no clear definition of massive transfusion [15]. The definition used was that of the Federal Statistical Office: more than 5 RBCs [14]. The rate of cell salvage usage was higher in transfused patients, at 3.4% (n = 90,433), compared with 1.1% (n = 200,826) in non-transfused patients.

**Figure 4:**
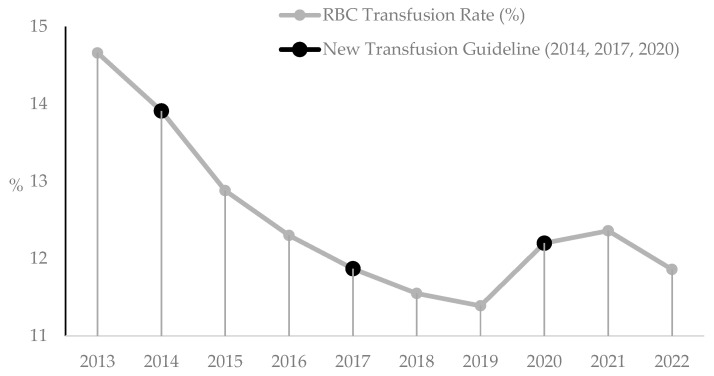
RBC transfusion rate in elderly hospitalized surgical patients in Germany (2013–2022) and implementation of new transfusion guidelines.

The percentage of transfused patients decreased from 14.7% (n = 306,318) in 2013 to 12.2% (n = 244,848) in 2020 (Figure 4). Detailed yearly data are provided in Table 4.

Multivariable logistic regression was used to assess the association between age and red blood cell transfusion while adjusting for sex (male vs. female), Elixhauser-Score, ICU-Stay (ICU vs. normal ward) and anemia (any type of anemia vs. no anemia). Anemia showed the strongest association with transfusion, with an adjusted OR of 14.93 (95% CI 12.31–18.15). A higher Elixhauser score was also associated with increased odds of transfusion, with an OR of 1.11 per score point. Female sex was associated with lower adjusted odds of transfusion compared with male sex. ICU stay was also associated with lower adjusted odds of transfusion compared with normal ward treatment (OR 0.24, 95% CI 0.24–0.24). The model showed good discrimination, with an Area under the curve (AUC) of 0.835 (Table 5).

## 4. Discussion

Clinicians are facing a growing population of elderly surgical patients. This study aimed to describe trends in transfusion practices in this growing, yet extremely heterogeneous, group over a 10-year period.

In this cohort of 21,143,317 surgical patients aged 65 years and older, we observed a transfusion rate of 12%. Most transfused patients (62.5%) were between 70 and 84 years old, while most non-transfused patients (69.2%) were between 65 and 79 years old. With increasing age, the odds ratio for transfusion increased continuously. The most common risk factor in both groups was essential hypertension. With the exception of obesity and chronic pulmonary disease, all analyzed risk factors were more prevalent in transfused patients. Compared with non-transfused patients, transfused patients had nearly four times the ICU admission rate, more than double the LOS, and longer ventilation times. Additionally, the rates of pneumonia, AKI, and all other examined complications were significantly higher in transfused patients. It is not possible to determine whether patients who receive blood transfusions were significantly more ill and therefore had higher complication rates, or whether these complications were a consequence of RBC transfusion. The Elixhauser score, which was over three times higher in transfused patients, suggests that the group of transfused patients was multimorbid.

Almost a third of the analyzed patients received RBCs (31.82%, n = 84,041) prior to surgery, whereas only approximately one-fifth of transfused patients received another blood product, such as platelets or fibrinogen.

It is well established that preoperative anemia is a risk factor for complications, longer LOS, and mortality [3], especially among elderly patients [7,16]. Patient blood management (PBM) is an effective program for identifying patients with treatable anemia and optimizing the patient’s own blood resources, thus reducing the need for RBC transfusion [17,18]. Our analysis revealed a decrease in the number of transfused patients between 2013 and 2020 by over 2% (Figure 4), which may be associated with the establishment of the PBM network in Germany [19]. There was a distinct increase in the RBC transfusion rate in 2020: it rose from 11.4% in 2019 to 12.2% in 2020 and 12.36% in 2021. This increase may be explained by the COVID-19 pandemic, as many critically ill patients were treated with liberal transfusion to provide oxygen carriers- [20]. Overall, 24.5% of all hospitalized COVID-19 patients between 1 January 2020 and 31 December 2021 in Germany were admitted to ICUs [21]. Raasveld et al. described 3643 adult patients admitted to ICUs in 30 countries from 2019 to 2022. In this study, 24.3% of ICU patients in Europe received at least one RBC transfusion [22].

The reported anemia rates, especially for iron deficiency anemia, were lower than those reported in the literature, which indicates anemia rates of 33% [16] to 43% [23] and iron deficiency anemia rates of around 12.6% [16] in elderly patients. This discrepancy may be due to underdiagnosis in our dataset. Most studies exclude patients undergoing minor surgeries. Specialized laboratory tests used to determine the type of anemia may be considered cost-prohibitive, especially for patients undergoing minor surgery. Furthermore, some hospitals do not routinely assess anemia or classify its subtype. Therefore, there is a high probability that anemia, and especially iron deficiency anemia, was underdiagnosed in our study population.

Due to the lack of Hb values, it is not possible to determine from this study whether the rising transfusion rates represented appropriate transfusions or whether overtransfusion may have occurred.

It is also not possible to determine when the analyzed complications occurred, no causal relationship can be established. It is possible that complications such as myocardial infarctions occurred in the context of anemia, which in turn triggered a transfusion. Similarly, anemic hypoxia is also a possible cause of AKI and thus explains the significantly higher rate among transfused patients.

Transfused patients likely differ from non-transfused patients, for example, in perioperative blood loss, surgical complexity, hemodynamic instability, preoperative anemia, or overall disease severity. Although the analysis adjusted for available variables such as age, sex, Elixhauser score, ICU admission, and anemia, important clinical information including hemoglobin levels, intraoperative blood loss, transfusion timing was not available in the administrative dataset. Therefore, postoperative complications may reflect the underlying clinical condition and indication for transfusion rather than the effect of transfusion itself. The observed associations between transfusion and complications should therefore be interpreted with caution.

The proportion of female patients in the transfused group was significantly higher than that in the non-transfused group (55.04% vs. 50.32%, respectively), even though the odds ratio of being transfused compared to male patients was 0.8 (95% CI 0.8–0.8). This difference may be partially explained by differing definitions of anemia for men and women. The World Health Organization (WHO) defines anemia as Hb < 13 g/dL in men and Hb < 12 g/dL in non-pregnant women [24]. This distinction may result in less preoperative optimization for women with lower hemoglobin levels, potentially increasing their risk of RBC transfusion [25]. Netz et al. analyzed 6516 patients, comparing hemoglobin values of <12.0 g/dL, 12.0–12.9 g/dL, and ≥13 g/dL among women, and found significantly higher transfusion rates, increased complications (e.g., pneumonia, AKI, and sepsis), and a longer LOS among female patients with Hb values of <13 g/dL [26]. The proportion of obese patients was significantly higher among non-transfused patients. The “obesity paradox” is widely discussed in the literature [27,28,29]. A possible explanation may be that greater metabolic reserves are available to meet the higher demands in the case of illness [27]. A correlation between obesity and iron deficiency is being investigated, which would not explain the lower proportion of obesity among non-transfused patients [30], but obesity is also associated with higher platelet counts, which may result in less perioperative bleeding [30]. Overall, the different rates may reflect unmeasured confounding. The data provided do not show causality. The proportion of patients suffering from chronic pulmonary disease was also significantly lower in transfused patients. This might be explained by a chronic state of hypoxia, which may trigger erythrocytosis [31] and a greater tolerance for anemia, although this cannot be proven using this dataset. The observed difference may also be influenced by coding bias, selection bias, or misclassification of comorbidities. As this study is based on retrospective registry data, these factors cannot be fully excluded and may have contributed to the large observed difference between the groups.

German transfusion guidelines changed during the study period (Figure 4). From 2008 on, it was characterized by a rather restrictive and individualized approach. RBC transfusion at Hb ≤ 6 g/dL, while transfusion at Hb > 10 g/dL was generally discouraged. For Hb values between 6 and 10 RBC transfusion was based on compensatory capacity, cardiovascular risk factors, and clinical signs of anemic hypoxia rather than Hb concentration alone. This framework was largely maintained in 2014. It continued to emphasize individualized decision-making and explicitly stated that Hb concentration alone is not an adequate measure of oxygen delivery.

The 2020 revision maintained this approach but introduced a relevant change by raising the general Hb threshold in the recommendation table from 6 to 7 g/dL. In addition, the 2020 guideline provided more differentiated recommendations for selected high-risk groups. In particular, for patients > 65 years in trauma and orthopedic surgery and for patients with relevant cardiovascular disease, transfusion was recommended at Hb < 8 g/dL, while for non-bleeding cardiac surgery patients, transfusion was recommended at Hb < 7.5 g/dL. The 2020 threshold changes may have contributed to stabilization or even increased transfusion rates in selected elderly surgical patients.

The most common complication in our study was acute kidney injury (AKI). The kidneys are highly sensitive to hypoxemia [32], and patients with anemia may have abnormal iron metabolism, which could result in iron-mediated oxidative kidney injury after RBC transfusion [32,33]. De Santo et al. [34] and Karkouti et al. [32] showed significantly higher postoperative AKI rates in patients with anemia [34] and in transfused patients [32]. Fowler et al. performed a meta-analysis including 949,445 patients and showed an association between anemia and AKI (OR 3.75, 95% CI 2.95–4.76; I(2) = 60%; *p* < 0.001) and infection (OR 1.93, 95% CI 1.17–3.18; I(2) = 99%; *p* = 0.01) [35].

In our analysis, patients with increasing age showed shorter LOS, ventilation times, and lower ICU admission rates. This could be due to the potential bias of analyzing both major and minor surgeries in our dataset. Most older patients may thus undergo minor or even outpatient surgery, which would reduce the significance of LOS as an outcome measure. Another reason for the decreasing ICU admission rate might be therapy limitations due to a patient directive or medical considerations in extremely frail patients [36]. These factors might result in shorter overall LOS and less invasive therapies.

The low proportion of other blood products compared to RBC transfusions suggests that these were not primarily administered in cases of massive bleeding, as the proportion of other blood products would have been higher in such cases. Another interpretation might be a transfusion futility threshold. Similarly, nearly one-third of the RBCs were already transfused prior to surgery. Almost 88% of the transfused patients received between 1 and 5 RBCs. It is possible that some of these transfusions could have been avoided if an anemia workup had been performed preoperatively. RBC transfusion decisions should always be made in the clinical context, not based on age alone.

Another potential bias to consider in our analysis is that transfused patients may have been more critically ill. This is reflected in the Elixhauser score, which was over three times higher in transfused patients.

Another interesting factor when examining older patients in particular could be delirium and its possible association with anemia and transfusion. Due to incomplete documentation and coding, the rates fluctuated too much over the analyzed time span to include them in this analysis. Van der Zanden et al. analyzed 415 patients aged between 65 and 102 years in a multicenter randomized controlled trial. They described a transfusion rate of 33.7% (n = 140) and a delirium rate of 32.5% (n = 115). Comparing patients with and without delirium, they found that the rates of anemia and blood transfusion were significantly higher in patients with delirium [37]. The transfusion rate was more than twice as high as that described in our study; this might be explained by the different Hb cutoff values used to determine transfusion eligibility, as this study was conducted in the Netherlands. Further studies should be performed to investigate a possible association between delirium and anemia, whether frailty influences RBC transfusion rates, and whether preoperative blood transfusions in patients undergoing major surgeries are a possible prognostic factor in older patients.

Overall, these data and their wide scope for interpretation clearly show the range and heterogeneity of aging. We observe severely ill, relatively young patients with many risk factors and high complication rates undergoing major surgeries. On the other hand, we see otherwise healthy older patients who undergo minor surgeries without relevant bleeding risk. It is important to assess each patient individually to determine whether they are high-risk. Our data show that this is not possible based on age alone. A more suitable approach seems to be the consideration of frailty. This multimodal approach reflects the diversity of age in several areas. It has already been shown that frailty is a predictor of negative health outcomes, such as increased mortality and a higher risk of hospitalization [1], and worse short- and long-term prognoses among older patients in the ICU [38].

### Limitations

This study has several limitations. First, the study’s retrospective nature and the use of secondary reimbursement data present inherent challenges. While reimbursement data correlate with medical cases in hospitals [39], the potential for over- or underrepresentation of conditions or events for reimbursement purposes cannot be entirely ruled out. Nonetheless, an increased incentive for accurate documentation exists, as hospital reimbursements are audited by the medical service of the health insurance funds. Parameters were selected based on their high medical relevance to minimize coding errors. The large sample size helps to mitigate the impact of any miscoded data. Data were collected in a structured and representative manner in accordance with the Declaration of Helsinki.

Since this study is based on registry data, associations can be identified, but no causal relationships can be inferred. Due to the large cohort, all correlations are statistically significant but may lack clinical relevance.

Based on data from the Federal Statistical Office, only surgical disciplines relevant to bleeding and RBC transfusion were analyzed. These disciplines were visceral surgery, cardiac surgery, thoracic surgery, gynecology, neurosurgery, oral and maxillofacial surgery, trauma and orthopedic surgery, urology, and vascular surgery. Furthermore, cases with more than one procedure were excluded from the analysis to exclude potential interactions between the procedures and their potential influence on transfusion practice. This led to a high number of excluded patients (n = 60,119,339). The inclusion of minor surgical procedures, such as those performed in ophthalmology, may have confounded the analysis. Nevertheless, even among the surgical disciplines analyzed, some procedures can be considered minor. It is not possible to differentiate between the different disciplines or between major and minor surgeries, which may represent a major confounding factor. Additional potential confounding factors, such as the distinction between elective and emergency surgeries, cannot be determined from the anonymized dataset. Key variables such as hemoglobin levels, clinical bleeding, or intraoperative blood loss are not coded, limiting conclusions regarding transfusion appropriateness. Furthermore, due to the nature of registry data, no clinical decision context (e.g., transfusion triggers) is reported.

As Hb values are not reported in this dataset, it is not possible to draw conclusions about rising transfusion rates in relation to anemia rates. The definition of massive transfusion is defined by the Federal Statistical Office. It reflects an administrative definition used and does not correspond to established clinical definitions. As a result, direct comparison with studies is limited.

Due to the exceptionally large sample size, *p*-values should be interpreted with caution, as even very small differences may reach statistical significance. Therefore, clinical interpretation was based primarily on absolute differences, adjusted odds ratios, and the consistency of observed trends rather than statistical significance alone. In addition, the regression models describe associations and do not allow causal inference. In particular, the lower adjusted odds of transfusion observed among ICU patients should not be interpreted as a protective effect of ICU admission.

Rather, this counterintuitive association could be the result of selection bias and the limitations of administrative data. Admission to the ICU represents a highly selected group of patients with a higher burden of disease, but the dataset does not include treatment goals, advanced directives, therapeutic restrictions, or the timing of the transfusion relative to admission to the ICU. It is also possible that comparable patients with similar disease severity and transfusion-requiring bleeding in the perioperative setting may have already died. Furthermore, it is possible that critically ill patients who were admitted to the ICU did not receive a red blood cell transfusion due to treatment restrictions or palliative care goals. It may also involve preemptive ICU admissions in elderly patients. Furthermore, admission to the ICU may serve as a post hoc marker influenced by numerous clinical factors, making the adjusted association susceptible to selection bias or confounding. Coding errors are also possible. Therefore, the finding regarding the ICU should be interpreted as an adjusted administrative association rather than as evidence that admission to the ICU reduces the need for transfusions.

This study provides a broad overview of transfusion practices in older patients but does not investigate the appropriateness of individual RBC transfusions, such as hemoglobin values or signs of ischemic hypoxia. Laboratory findings or medication data are not coded for reimbursement and thus were unavailable for analysis. A notable strength of this study is its scale, as it is the largest of its kind. The use of high-quality nationwide data over a period of 10 years provides an unbiased analysis of the current state of care in Germany.

## 5. Conclusions

Our study reveals that increasing age is associated with a higher chance of being transfused. Furthermore, as elderly patients show substantial heterogeneity, the main goal in treating this population should be to identify their needs, optimize their own resources, and not accept anemia as a side effect of aging. Individualized transfusion protocols could be helpful for the rational use of blood products in this vulnerable group. Further studies should aim to identify specific risk factors in elderly patients undergoing surgery.

## Figures and Tables

**Table 1:** Demographics of transfused elderly hospitalized surgical patients in Germany (2013–2022) according to age group.

Age Groups (Years)	65–69		70–74		75–79		80–84		85–89		90–94		95–110		*p*- Value
	**n**	**%**	**n**	**%**	**n**	**%**	**n**	**%**	**n**	**%**	**n**	**%**	**n**	**%**	
Total patients	327,380	12	436,264	17	607,324	23	605,436	23	422,128	16	195,916	7	46,160	2	<0.0001
Female	139,528	43	200,564	46	308,086	51	345,719	57	276,158	65	145,680	74	37,641	82	<0.0001
ICU	74,673	23	94,445	22	124,039	20	113,378	19	73,205	17	33,347	17	7840	17	<0.0001
Age; years, median (Q1; Q3)	67 (66; 68)		72 (71; 73)		77 (76; 78)		82 (81; 83)		87 (86; 88)		91 (90; 93)		96 (95; 98)		<0.0001
Elixhauser-score; n, median (Q1; Q3)	10 (4; 18)		10 (4; 17)		10 (5; 17)		11 (5; 17)		10 (5; 17)		10 (5; 17)		10 (5; 15)		<0.0001
Length of stay; hours, median (Q1; Q3)	367 (238; 651)		365 (240; 634)		366 (242; 622)		365 (239; 601)		358 (234; 569)		332 (217; 520)		299 (199; 468)		<0.0001
Ventilation; hours, median (Q1; Q3)	95 (25; 338)		82 (24; 315)		74 (22; 283)		64 (19; 225)		48 (14; 158)		31 (9; 87)		24 (7; 61)		<0.0001
**Anaemia**	**n**	**%**	**n**	**%**	**n**	**%**	**n**	**%**	**n**	**%**	**n**	**%**	**n**	**%**	
Iron deficiency anemia	23,584	7	31,657	7.3	46,220	8	49,907	8	33,055	8	12,437	6	2301	5	<0.0001
Vitamin B12, folic acid, any other dietary anemia	4032	1	4960	1.1	7442	1	8583	1	6341	2	2884	1.5	637	1	<0.0001
Any other form of anemia	295,440	90	393,694	90.2	547,952	90	544,886	90	382,818	91	181,003	92	43,366	94	<0.0001
Anemia due to acute bleeding	247,952	76	337,869	77.5	480,525	79	484,838	80	349,427	83	169,889	87	41,501	90	<0.0001
**Complications**	**n**	**%**	**n**	**%**	**n**	**%**	**n**	**%**	**n**	**%**	**n**	**%**	**n**	**%**	
Stroke	5500	2	7048	2	9268	2	8048	1	4861	1	1834	1	346	1	<0.0001
CPR	11,879	4	14,950	3	20,078	3	17,925	3	10,357	3	3463	2	593	1	<0.0001
Pneumonia	44,402	14	55,672	13	75,127	12	72,580	12	49,219	12	22,087	11	5359	12	<0.0001
AKI	57,284	18	73,355	17	105,377	17	111,348	18	76,680	18	33,844	17	8135	18	<0.0001
RRT	29,605	9	34,469	8	42,910	7	33,907	6	14,186	3	2725	1	231	1	<0.0001
Complication after transfusion	3774	1	4829	1	6711	1	6324	1	4090	1	1737	1	332	1	<0.0001
Pulmonary embolism	4382	1	5157	1	6363	1	6091	1	3790	1	1434	1	269	1	<0.0001
Myocardial infarction	7007	2	9769	2	14,113	2.	13,896	2	9552	2	3904	2	783	2	<0.0001

CPR: Cardiopulmonary Resuscitation. AKI: Acute kidney injury. RRT: renal replacement therapy.

**Table 2:** Demographics of elderly hospitalized surgical patients with or without red blood cell (RBC) transfusion in Germany (2013–2022).

	with RBC Transfusion		Without RBC Transfusion	
	n	%	n	%
Total patients	2,640,608		18,502,709	
Female	1,453,376	55	9,310,912	50
ICU	521 927	20	922,756	5
Age; years, median (Q1; Q3)	79 (73; 85)		76 (70; 81)	
Elixhauser-score; n, median (Q1; Q3)	10 (5; 17)		3 (0; 8)	
Length of stay; hours, median (Q1; Q3)	362 (238; 600)		150 (74; 263)	
Ventilation; hours, median (Q1; Q3)	70 (20; 260)		30 (8; 112)	
**Age groups (y)**				
65–69	327,380	12	4,006,977	22
70–74	436,264	17	4,225,018	23
75–79	607,324	23	4564,518	25
80–84	605,436	23	3,392,009	18
85–89	422,128	16	1,665,638	9
90–94	195,916	7	551,307	3
95–110	46,160	2	97,242	1
**Anaemia**				
Iron deficiency anemia	199,161	8	285,435	2
Vitamin B12, folic acid, any other dietary anemia	34,879	1	56,878	0.3
Any other form of anemia	2,389,159	91	909,765	5
Anemia due to acute bleeding	2,112,001	80	551,151	3
**Complications**				
Stroke	36,905	1	67,466	0.4
CPR	79,245	3	76,224	0.4
Pneumonia	324,446	12	386,876	2
AKI	466,023	18	738,740	4
RRT	158,033	6	227,968	1
Complication after transfusion	27,797	1	n.a.	n.a.
Pulmonary embolism	27,486	1	47,396	0.3
Myocardial infarction	59,024	2	69,813	0.4

RBC: red blood cell; CPR: Cardiopulmonary resuscitation, AKI: Acute kidney injury. n.a.: not applicable, as these group did not recieve RBCs.

**Table 3:** Characteristics of RBC and coagulants administration among elderly hospitalized surgical patients in Germany (2013–2022).

	with RBC Transfusion		Without RBC Transfusion	
**RBC administration prior to surgery**	n	%	n/a	
Yes	84,014	32	n/a	
No	1,799,849	68	n/a	
undefined	619	0.02	n/a	
**Blood products administered**	n	%	n	%
RBCs	2,640,608	100	0	0
Platelets	106,564	4	27,094	0.2
Fresh Frozen Plasma	233,675	4	27,039	0.2
Prothrombin complex concentrate	152,391	6	105,059	0.6
Fibrinogen	96,010	4	16,438	0.1
Massive blood transfusion	76,005	3	0	0
Cell salvage	90,433	3	200,826	1

n/a: not applicable.

**Table 4:** The use of RBCs per year in elderly hospitalized surgical patients in Germany (2013–2022).

	Total		RBC Transfusion	
Year	n	%	n	%
2013	2,089,608	10	306,318	15
2014	2,116,587	10	294,413	14
2015	2,127,474	10	274,087	13
2016	2,158,388	10	265,412	12
2017	2,168,011	10	257,254	12
2018	2,174,269	10	251,087	12
2019	2,207,750	10	251,466	11
2020	2,007,317	9	244,848	12
2021	2,011,032	10	248,628	12
2022	2,082,881	10	247,095	12

Total: proportion of patients each year, RBC Transfusion n: count of patients receiving at least one RBC: The % represents the proportion of all patients in this year. RBC: Red blood cell.

**Table 5:** Logistic regression models of the chance of red blood cell use in elderly patients according to age groups, sex (male vs. female), Elixhauser-Score, ICU-Stay (ICU vs. normal ward) and anemia (any type of anemia vs. no anemia) in surgical patients in Germany (2013–2022); For the estimation of the odds ratio for transfusion among age groups, patients aged < 65 were used as the reference group for this specific analysis, including this otherwise excluded group from the primary cohort.

	Odds-Ratio	95% CI
Age 65–69	1.5	1.5–1.6
Age 70–74	1.7	1.7–1.8
Age 75–79	1.9	1.9–1.9
Age 80–84	2.2	2.2–2.2
Age 85–89	2.7	2.7–2.7
Age 90–94	3.7	3.6–3.7
Age 95–110	5	4.9–5.1
Female sex	0.8	0.8–0.8
Elixhauser-Score	1.1	1.1–1.1
ICU-Stay	0.2	0.2–0.2
Anemia	15	12.3–18.2

ICU = Intensive care Unit, CI = Confidence Interval.

## Data Availability

In Germany, hospitals are legally required to report relevant diagnoses using the International Statistical Classification of Diseases and Related Health Problems (ICD) codes and the International Statistical Classification of Procedures (OPS) codes. According to §21 of the German Hospital Finance Law (KHG), all hospitals must submit anonymized data to the Institute for Hospital Remuneration (InEK) for the ongoing development of the Diagnosis-Related Group (DRG) system. This data is subsequently transferred by InEK to the Federal Statistical Office (DESTATIS), where it is made accessible to researchers under a signed data usage contract. The reporting of ICD-10 and OPS codes, along with the dependency of reimbursement on this data, ensures comprehensive recording of all treatments in German hospitals. The data are stored locally by the German Federal Statistical Office. Individual patient and hospital identifiers were not accessible to the authors.

## Abbreviations

The following abbreviations are used in this manuscript:

AKI	Acute Kidney Injury
CGA	Comprehensive Geriatric Assessment
CPR	Cardiopulmonary Resuscitation
DRG	Diagnosis-Related Group
FFP	Fresh Frozen Plasma
GDP	General Data Protection Regulation
ICD	International Statistical Classification of Diseases and Related Health Problems
ICU	Intensive Care Unit
InEK	Institute for Hospital Remuneration
KHG	German Hospital Finance Law
LOS	Length of Stay
OPS	International Statistical Classification of Procedures
OR	Odds Ratio
PCC	Prothrombin Complex Concentrate
PPI	Proton Pump Inhibitor
RBC	Red Blood Cell
RRT	Renal Replacement Therapy
WHO	World Health Organization

## Appendix A

**Table A1:** Classification of Diseases and Related Health Problems (ICD) codes and the International Statistical Classification of Procedures (OPS) codes included in our analysis. CPR: Cardiopulmonary resuscitation. PE: Pulmonary Embolism.

Diagnosis	OPS Codes	ICD-10
CPR	8-771, 8-772, 8-779	8-771, 8-772, 8-779
Essential hypertension		I10.-
Congestive heart failure		I09.9, I11.0, I13.0, I13.2, I25.5, I42.0, I42.5–I42.9, I43.x, I50.x, P29.0
Obesity		E66.-
Red blood cells	8-800.c	
Platelets	8-800.6, 8-800.d, 8-800.f, 8-800.g, 8-800.h, 8-800.j, 8-800.k, 8-800.m, 8-800.n	
Fresh Frozen Plasma	8.812.6–8.812.8	
Prothrombin complex concentrate	8-812.5	
Fibrinogen	8-810.j	
Massive blood transfusion	8-800.1	
Anticoagulation therapy		Z92.1
Stroke		I63, I64
Pneumonia		J12.-, J13, J14, J15.-, J16.-, J17.x, J18.-, U69.00, J11.0
Renal replacement therapy	8-853., 8-854., 8-855.	
Complication after transfusion		I80.x
PE		I26.x
Myocardial infarction		I21., I22., I24.
AKI		I12.0, I13.1, N18.x, N19.x, N25.0, Z49.0Z49.2, Z94.0, Z99.2
Iron deficiency anemia		D50.x
Vitamin B12, folic acid, any other dietary anemia		D51.x–D53.x
Any other form of anemia		D55.x–D64.x
Anemia due to acute bleeding		D62

**Table A2:** Risk factors of transfused elderly hospitalized surgical patients in Germany (2013–2022) according to age groups.

Age Groups (Years)	65–69		70–74		75–79		80–84		85–89		90–94		95–110		*p*-Value
	n	%	n	%	n	%	n	%	n	%	n	%	n	%	
**Risk factors**															
Essential hypertension	183,775	56.1	261,390	59.9	378,343	62.3	385,792	63.7	270,749	64.1	126,802	64.7	29,682	64.3	<0.0001
Anticoagulation therapy	45,904	14.0	74,107	17.0	121,709	20.0	140,287	23.2	98,151	23.3	39,342	20.1	7303	15.8	<0.0001
Congestive heart failure	77,594	23.7	112,746	25.8	173,624	28.6	188,307	31.1	138,393	32.8	64,496	32.9	14,721	31.9	<0.0001
Cardiac arrhythmias	93,870	28.7	150,818	34.6	243,449	40.1	263,639	43.6	186,611	44.2	80,853	41.3	16,541	35.8	<0.0001
Chronic pulmonary disease	47,206	14.4	62,636	14.4	81,060	13.4	72,870	12.0	43,925	10.4	16,830	8.6	3135	6.8	<0.0001
Metastatic cancer	31,731	9.7	35,523	8.1	38,039	6.3	29,110	4.8	13,770	3.3	3790	1.9	437	1.0	<0.0001
Solid tumor without metastasis	29,840	9.1	37,461	8.6	43,479	7.2	36,895	6.1	20,141	4.8	6601	3.4	1030	2.2	<0.0001
Alcohol abuse	20,715	6.3	15,866	3.6	12,078	2.0	5883	1.0	1693	0.4	319	0.2	34	0.1	<0.0001
Obesity	37,562	11.5	44,084	10.1	50,701	8.4	37,090	6.1	15,494	3.7	3663	1.9	494	1.1	<0.0001

**Table A3:** Risk factors of elderly hospitalized surgical patients with or without red blood cell transfusion in Germany (2013–2022).

	with RBC Transfusion		Without RBC Transfusion	
	n	%	n	%
**Risk factors**				
Essential hypertension	1,636,533	61.98	11,088,807	59.93
Anticoagulation therapy	526,803	19.95	2,767,187	14.96
Congestive heart failure	769,881	29.16	2,201,409	11.9
Cardiac arrhythmias	1,035,781	39.23	4,173,921	22.56
Chronic pulmonary disease	327,662	0.47	1,646,280	8.9
Metastatic cancer	152,400	5.77	626,178	3.38
Solid tumor without metastasis	175,447	6.64	838,251	4.53
Alcohol abuse	56,588	2.14	148,229	0.8
Obesity	189,088	7.16	1,507,069	8.15

**Table A4:** Timepoints and Number of Red Blood Cell administration in elderly hospitalized surgical patients in Germany (2013–2022), RBC: Red Blood Cell).

	with RBC Transfusion		Without RBC Transfusion	
**Timepoint of RBC administration**				
Admission to surgery ; hours, median (Q1;Q3)	28.73 (16.6; 117.43)		22.48 (4.28; 45.52)	
Admission to RBC ; hours, median (Q1;Q3)	61.02 (20.6; 165.4)			
Surgery to RBC ; hours, median (Q1;Q3)	6.7 (−5.8; 60.6)			
RBC prior to surgery ; hours, median (Q1;Q3)	−35.3 (−122.1; −9.34)			
RBC after surgery ; hours, median (Q1;Q3)	32.6 (5.7; 99.9)			
**Number of RBCs**	n	%		
1–5	2,323,148	87.98		
6–10	244,272	9.25		
11–15	46,684	1.77		
16–23	20,582	0.78		
24–31	5402	0.2		
32–39	1846	0.07		
40–47	725	0.03		
48–55	329	0.03		
56–63	140	0.01		
64–71	90	0		
72–79	42	0		
